# Nurses' Perceptions of Their Non‐Technical Skills in Recognising and Responding to Clinical Deterioration in Hospital Settings: A Systematic Review

**DOI:** 10.1111/jocn.70497

**Published:** 2026-08-13

**Authors:** Darlene Archer, Amineh Rashidi, Rajbeer Kaur, Allison Roderick, Olivia Gallagher

**Affiliations:** ^1^ School of Nursing & Midwifery Adelaide University Adelaide South Australia Australia; ^2^ School of Nursing & Midwifery Edith Cowan University Joondalup Western Australia Australia; ^3^ School of Nursing Curtin University Bentley Western Australia Australia; ^4^ SwanCare Bentley Western Australia Australia; ^5^ School of Health & Clinical Sciences University of Western Australia Crawley Western Australia Australia

**Keywords:** clinical deterioration, hospital, non‐technical skills, nurses, systematic review

## Abstract

**Aim:**

To understand nurses' perceptions of non‐technical skills in recognising and responding to clinical deterioration in hospital settings.

**Design:**

A mixed methods systematic review was undertaken, guided by the Joanna Briggs Institute Manual for Evidence Synthesis.

**Methods:**

A systematic search of the primary research literature published between 2012 and October 2025 was undertaken. A convergent integrated approach to synthesis, without meta‐analysis, was undertaken.

**Data Sources:**

The MEDLINE, CINAHL, Scopus, PsychInfo and Embase electronic databases were searched. Reference lists of full‐text articles retrieved were reviewed for additional data sources.

**Results:**

Twenty‐four primary studies were included. Narrative synthesis resulted in six main themes: (1) situational awareness, (2) effective teamwork and communication, (3) leadership and culture, (4) intuition, (5) level of experience and (6) fear of retribution. Across studies, supportive team culture and strong communication facilitated recognition and escalation, whereas inexperience and fear of criticism delayed action.

**Conclusions:**

Early recognition and response to a clinical deterioration is beyond simply detecting physiological parameters. The process of recognition and response to a deterioration is enabled due to non‐technical skills. In situations where the team has effective and positive team culture, where there is psychological safety, where all levels of expertise are given a voice without fear of retribution, everyone is safe to raise the alarm. Strengthening these skills and supporting junior nurses may improve escalation practice.

**Implications for the Profession and/or Patient Care:**

The implementation of early warning scores or rapid response systems is a well‐developed mechanism to improve outcomes for deteriorating patients. Unfortunately, there has been less emphasis and value placed on development of non‐technical skills in the recognition and response to clinical deterioration. Organisations and professions need to mitigate barriers to the implementation of nurses' non‐technical skills to improve patient outcomes.

**Impact:**

- What problem did the study address?
  ○ A lack of current synthesised evidence of nurses' non‐technical skills in the recognition of, and response to, clinical deterioration.
- What were the main findings?
  ○ Organisational cultures that devalue less experienced nurses and non‐technical skills, risk delays in recognition and response to clinical deterioration.
- Where and on whom will the research have an impact?
  ○ Patients, health care professionals and health care delivery may benefit from integration of non‐technical skills into deterioration response frameworks, implemented within psychologically safe organisational cultures.

**Reporting Method**

Preferred Reporting Items for Systematic Reviews and Meta‐Analyses (2020).

**Patient or Public Contribution**

No patient or public contribution. This review received no financial support.

**Trial Registration**

The protocol for this review was registered with PROSPERO (CRD42022327788) (crd.york.ac.uk/PROSPERO/display_record.php?RecordID=327788)

## Summary

- What does this paper contribute to the wider global clinical community?
  ○ This review synthesises the latest evidence regarding nurses' non‐technical skills in the recognition of, and response to, clinical deterioration.
  ○ The review identified that nurses' non‐technical skills could have a positive impact on the recognition of, and response to, clinical deterioration; yet there are barriers to their effective implementation.
  ○ This review highlights the recognition of, and response to, clinical deterioration can potentially improve outcomes through the valuing of nurses' non‐technical skills.

## 1 Introduction

Clinical deterioration of a patient is defined as sudden changes in a patient's condition that could result in serious adverse events and is often preceded by observable physiological and clinical abnormalities (Petersen et al. 2017). Adverse events resulting from ineffective recognition and escalation of clinical deterioration include prolonged hospital stays, in‐hospital cardiac arrest, unanticipated admission to an intensive care unit or mortality, which are often preventable (Walker et al. 2021). Poor recognition or response to clinical deterioration is also a contributing factor to financial burden on healthcare systems (Curtis et al. 2021).

Clinical deterioration remains a major international patient safety concern, with delayed recognition and escalation contributing to preventable adverse outcomes, including cardiac arrest, unplanned intensive care admission and death (Piasecki et al. 2023). Rapid response systems and early warning scores have been widely introduced to support timely detection and escalation; however, their effectiveness depends not only on physiological triggers and escalation protocols but also on nurses' non‐technical skills, including situational awareness, communication, teamwork, leadership and clinical judgement (Zhang et al. 2024).

Although local standards such as Australia's Recognising and Responding to Acute Deterioration Standard provide an important policy framework (Australian Commission on Safety and Quality in Health Care 2021), the challenge of recognising and responding to deterioration is shared across health systems internationally (Hall et al. 2020). To facilitate early identification of clinical deterioration, the use of rapid response systems (RRS) and early warning scores (EWS) is widespread in hospitals in the United Kingdom, Australia and the United States of America. These tools guide health practitioners to recognise and respond appropriately to prevent serious adverse events (Petersen et al. 2017). Despite a growing body of literature on rapid response systems, less is known about how nurses' non‐technical skills specifically shape recognition and escalation practices in acute care (Australian Commission on Safety and Quality in Health Care 2021). This review therefore aims to synthesise the available evidence on nurses' perceptions of their non‐technical skills in recognising and responding to clinical deterioration in hospital settings.

Nurses, as frontline healthcare workers, play an essential role in recognising and responding to clinical deterioration (Bingham et al. 2020; Considine and Currey 2015; Langkjaer et al. 2021; Mølgaard et al. 2022). Delays to escalation could prolong time to treatment for deteriorating patients and increase their chances of mortality (Walker et al. 2021). During the last decade, many studies focused on identifying barriers in initiating a rapid response system and factors that influence recognition and escalation (Labrague et al. 2019; Mølgaard et al. 2022). Nurses' technical skills are linked with ability to identify physiological parameters of clinical deterioration, however, the importance of nurses' non‐technical skills in this process is not emphasised (Bingham et al. 2020; Labrague et al. 2019; Mølgaard et al. 2022). Non‐technical skills specific to health care professions are described by Peddle et al. (2018) as situational awareness, decision making, communication, teamwork, leadership, managing stress and coping with fatigue. The terminology non‐technical is however misleading. Non‐technical suggests simple, easy or soft skills. Non‐technical skills are highly complex and represent the cognitive and social skills that enable human systems to function (McCarthy and Blumenthal 2006). For nurses, as the largest workforce across acute health care settings, this is crucial.

Evidence from systematic reviews indicates that rapid response systems and rapid response teams are associated with improved patient outcomes when they are well implemented, mature, and supported by dedicated education, proactive rounding and clear activation processes. Although these systems are designed to support early recognition and escalation of clinical deterioration, their effectiveness depends not only on trigger thresholds but also on the clinical judgement, communication and teamwork of frontline staff (Daniele et al. 2011).

Peddle et al.'s (2018) study undertook a qualitative comparative analysis to identify what non‐technical skills competencies are addressed by Australian standards documents for 11 health care professions. The competency statements included for analysis were those that contained consistent behavioural and measurable statements. Varying elements across the same six categories were identified for nurses (Table 1). This comparison reflects how the competencies are articulated in the source document and does not imply that these skills are unique to one nursing group.

**TABLE 1:** Categories and elements of clinical deterioration competencies (Peddle et al. 2018).

Category	Elements for the registered nurse	Elements for the enrolled nurse
Situation awareness	Gathering information	Gathering information Interpreting information Anticipating future states
Decision‐making	Outcome review	Situation assessment Outcome review
Communication	Send information clearly and concisely	Send information clearly and concisely Include context and intent during information exchange Identify and address barriers to communication
Team working	Exchange information Coordinate activities Feedback Backing up behaviours Team self‐awareness	Exchange information Coordinate activities Backing up behaviours Fostering team interdependence
Leadership	Use authority Maintain standards Plan and prioritise Manage workload and resources	Use authority Maintain standards Plan and prioritise Manage workload and resources
Managing stress	Recognise symptoms and effects Implement coping strategies	Implement coping strategies

Peddle et al.'s (2018) analysis of non‐technical skills was adopted as a framework for defining non‐technical skills of nurses in this study. Whilst identification was specific to professional groups, comparison and contrast between professional groups recognises the importance of context to interdisciplinary practice in the recognition and response to clinical deterioration of patients in hospital settings.

## 2 The Review

### 2.1 Aim

The aim of the systematic review was to understand nurses' perceptions of their non‐technical skills in recognising and responding to clinical deterioration in hospital settings.

### 2.2 Methods

#### 2.2.1 Design

The Joanna Briggs Institute Manual for Evidence Synthesis guided this mixed method systematic review (Lizarondo et al. 2020). Non‐technical skills in clinical deterioration reflect human factors lending themselves to both behavioural and objective competency elements. In the context of clinical deterioration, non‐technical skills include the cognitive and social skill or competence that support safe recognition and response. This phenomenon therefore inherently captures qualitative and quantitative aspects of competence/skill. Therefore, a mixed‐methods systematic review was chosen as the most appropriate methodological approach for this review as it would enable a more comprehensive understanding of the phenomenon by synthesising the breadth of qualitative and quantitative data on this topic. A mixed‐method review enables an overarching understanding that can best inform the development of best‐practice recommendations (Stern et al. 2021). The review included qualitative, quantitative and mixed methods primary studies. The protocol for this review was registered with PROSPERO (CRD42022327788). The protocol and manuscript were developed according to the Preferred Reporting Items for Systematic Reviews and Meta‐Analysis guidelines (File S1).

#### 2.2.2 Search Methods

The search strategy was developed through identification of the participants (nurses), phenomenon of interest (non‐technical skills in clinical deterioration) and context (acute care settings) under investigation. First, an initial limited search of a university library search engine was undertaken followed by analysis of the text words contained in the title and abstract and the index terms used to describe the articles. The Participant, Phenomenon of (I)nterest, Context and Outcomes (PICO) framework was utilised to identify key words and search terms as outlined in Table 2. Non‐technical skills were defined based upon Peddle's conceptual analysis of non‐technical skills. The search terms were extrapolated to include ‘non‐clinical’ along with each of the components of Peddle's conceptual analysis of non‐technical skills (situational awareness, decision making, teamwork, leadership, communication, managing stress, coping with fatigue). The Boolean operator AND was applied between PICO components, and the operator OR applied within component search terms. Databases searched included MEDLINE, CINAHL, Scopus, PsychInfo and Embase (File S2). Search results obtained on June 2, 2022, and updated on February 7, 2024 and then October 27, 2025, were exported into Endnote and automatic duplication removal completed, followed by manual duplication removal.

**TABLE 2:** PICO framework of search terms.

PICO	Key words	Search terms
Participant	Nurses	Nurs* Enrolled nurs* Registered nurs*
Phenomenon of (i)nterest	Use of non‐technical skills	Non‐clinical skills Non‐technical skills Situational awareness Decision making Teamwork Leadership Communication Managing stress Coping with fatigue
	Recognising and responding to clinical deterioration	Clinical deterioration Deteriorat* Early warning Rapid response Escalati*
Context	Acute care settings	Acute care In‐patient Hospital Health service Health care facility Tertiary
Outcomes	Perceptions	Perception Belief Experience Outcome

* Symbol for truncation of search terms within databases.

#### 2.2.3 Inclusion and/or Exclusion Criteria

Inclusion criteria were original research investigating nurses' perceptions of their non‐technical skills in recognising and responding to clinical deterioration. Qualitative, quantitative and mixed‐methods studies were included. Studies including other health care professionals were included if results presented included the perceptions of nurses. Articles were published in English since 2012, accessible in full text (Table 3).

**TABLE 3:** Inclusion and exclusion criteria.

Inclusion criteria	Exclusion criteria
Nurse participants	Studies did not include nurse participants or where data from nurse participants was not reported separately from other participant groups
Non‐technical skills in recognising and responding to clinical deterioration (situational awareness, decision making, teamwork, leadership, communication, managing stress, coping with fatigue)	Studies investigating technical skills only
Primary studies	Reviews, conference proceedings, discussion papers or opinion pieces
Published in English	Not published in English
Published since 2012	Published before 2012

#### 2.2.4 Search Outcome

A total of 4010 records were retrieved for screening. Study selection was undertaken within the JBI SUMARI application. Title and abstract screening were undertaken independently by two reviewers. Conflicts were discussed and resolved during team meetings. Full‐text articles included were reviewed independently by two reviewers. Conflicts were discussed at team meetings and resolution achieved by consensus of the team. Reference lists of full‐text articles included were reviewed for article titles that may meet inclusion criteria. The process of title and abstract, and full‐text screening was repeated for these articles and 44 studies were excluded following full‐text review, with a total of 24 articles sourced for quality appraisal. The selection process is detailed in the PRISMA flow diagram (Figure 1).

**FIGURE 1:**
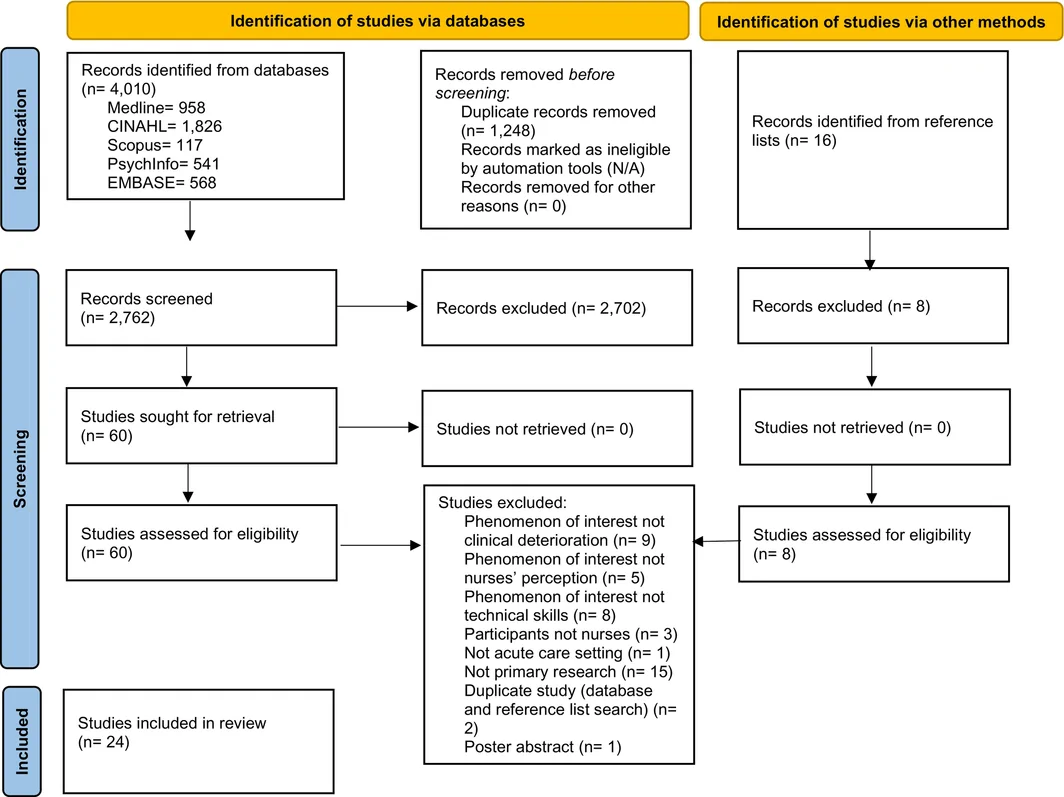
PRISMA flow diagram (http://www.prisma‐statement.org/). [Colour figure can be viewed at wileyonlinelibrary.com]

#### 2.2.5 Quality Appraisal

Critical appraisal of each study was conducted independently by two reviewers using JBI tools appropriate to each study design and the Mixed Methods Appraisal Tool (2018) for mixed‐methods studies. Appraisals were compiled and discussed to resolution. The included studies demonstrated predominantly high methodological quality, with most receiving ‘yes’ responses across criteria. Isolated ‘no,’ ‘unclear,’ or ‘can't tell’ ratings (e.g., researcher reflexivity in Anton et al. 2021; Ruiz et al. 2024; measurement consistency in Ekwantoro and Rusca 2020; Saunders et al. 2021) were noted but did not indicate systemic weaknesses warranting exclusion. As per JBI guidance (Lizarondo et al. 2020), all studies meeting inclusion criteria were retained in the synthesis to avoid bias from quality‐based exclusion. Quality appraisal is provided in File S3.

#### 2.2.6 Data Extraction

Data from included articles was extracted into a pre‐formed Excel spreadsheet, reporting the following study characteristics: author, year, methodology, country, aims, design, methods, population and sample size, measures, findings/results and limitations. Where study participants included other health professions, only data related to nurses' perceptions of their non‐technical skills were extracted. Data extraction was confirmed by a second reviewer.

#### 2.2.7 Synthesis

A convergent integrated synthesis was conducted due to the nature of the review question being able to be answered by both qualitative and quantitative methods. The convergent approach combines extracted qualitative and quantitative data by transforming it into one set of data. Quantitative data (including the quantitative data from mixed methods studies) were qualitised. Qualitising quantitative data was most appropriate as most of the extracted data were qualitative. There is also a greater risk of error in transforming qualitative data into numerical values (Stern et al. 2021). The synthesis process first entailed two reviewers (DA and AR) transforming quantitative data into textual descriptions. Quantitative data were qualitised by translating numerical findings into narrative descriptions that captured their meaning and direction in relation to the review question. This process was interpretive and descriptive, with all transformation decisions recorded in a coding table (exemplar provided in Table 4). The pooled data were then repeatedly reviewed and categorised by the two reviewers, adopting a deductive analysis approach aligned to the components of non‐technical skills as described by Peddle et al. (2018). Coding occurred in two rounds: the first generated initial codes, and the second refined these into broader categories. Disagreements were resolved by a third reviewer (OG), and all decisions were documented in discussion logs. Glaser and Straus' (2017) constant comparison method saw coding compared and contrasted. This approach is based on the idea that themes represent the ways in which texts are either similar or different from each other Glaser and Strauss (2017). The research team then reviewed these themes against the original protocol, to ensure these themes represented the desired aim of the project. An example of the synthesis process is provided in Table 5.

**TABLE 4:** Audit trail qualitising quantitative data.

Study (methodology)	Quantitative finding (as reported)	Qualitised text (textual transformation)	Subtheme	Synthesis theme
Orique et al. (2019) USA Quantitative	Nurses' tendency to classify patient cues as signals of clinical deterioration was significantly different (*p* < 0.05) across medical‐surgical subspecialties. Nurses in the acute postsurgical unit showed a significantly lower tendency compared with renal, oncology, and observation units	Nurses in specialties where physiological variability is expected (e.g., postoperative care) were less inclined to classify subtle changes as deterioration signals. The clinical environment shapes the criteria nurses apply when interpreting cues, influencing situational awareness	Situational awareness—cue interpretation; knowing the patient	Theme 1: Situational awareness
Hart et al. (2014) USA Quantitative	Self‐confidence was positively associated with leadership abilities in nurses responding to acute deterioration events. Nurses with stronger perceived leadership reported higher confidence in clinical reasoning and decision‐making during deterioration	Nurses who perceived themselves as leaders reported greater self‐confidence in recognising and responding to deterioration. Leadership functioned as a self‐assessed capacity to act decisively, not merely a hierarchical role	Leadership—self‐confidence; decision‐making under pressure	Theme 3: Leadership and culture
Ekwantoro and Rusca (2020) Indonesia Quantitative	Nurses' knowledge and clinical experience were positively correlated with capacity to recognise and respond to clinical deterioration. Less experienced nurses demonstrated reduced ability to detect subtle changes	More experienced nurses were better equipped to detect and respond to deterioration using non‐technical skills. Less experienced nurses relied primarily on physiological parameters, consistent with qualitative accounts of novice nurses' limited NTS repertoire	Experience versus inexperience; intuition—NTS development over time	Theme 4: Intuition Theme 5: Experience versus inexperience
Azimirad et al. (2022) Finland Quantitative	Effective leadership was correlated with embedding a positive organisational culture, which motivated nurses to support less experienced colleagues and engage with deterioration recognition without fear, strengthening self‐confidence	Leadership practices were associated with increased nurse confidence in managing deterioration. A culture fostered by effective leadership enabled nurses to focus on early recognition, support peers and provide high‐level care—reducing delays linked to fear or self‐doubt	Leadership—positive culture; managing stress; empowerment	Theme 3: Leadership and culture
Langkjaer et al. (2021) Denmark Mixed methods (quantitative component only)	Questionnaire data indicated nurses' reliance on senses, clinical gaze and non‐physiological cues varied with experience; experienced nurses reported higher use of non‐technical cues relative to vital signs monitoring	Experienced nurses were more likely to draw on their senses and clinical gaze—rather than physiological parameters alone—to detect deterioration. The clinical gaze represents an acquired NTS that develops with years of practice rather than initial training	Situational awareness—clinical gaze; intuition; experience versus inexperience	Theme 1: Situational awareness Theme 4: Intuition Theme 5: Experience versus inexperience
McNeill et al. (2019) USA Mixed methods (quantitative component only)	Questionnaire data indicated nurses reported varying levels of communication competence during deterioration; discrepancies were found between nurses' self‐reported communication and patients' perceptions of that communication	Nurses varied in their perceived communication skills during deterioration events. Gaps between nurses' self‐assessment and patient perceptions suggest a potential blind spot in nurses' situational awareness of how their communication is received	Communication—clarity; situational awareness of communication impact	Theme 2: Teamwork and communication

**TABLE 5:** Exemplars of data synthesis.

Study	Study coding	Exemplar	Theme
Exemplar 1: Anton et al. (2021)	Clinical judgement, clinical decision‐making, teamwork, experience verses inexperience, engagement, nursing presence, situational awareness	‘Being with a patient face to face, I can assess them no matter what I am doing. Just being person to person, it's an important thing…’ (Anton et al. 2021, 15) Subthemes—engagement, situational awareness, nursing presence	Situational awareness
Exemplar 2: Langkjaer et al. (2021)	Intuition, communication, experience versus inexperience, clinical gaze, senses	‘Just to touch and feel the patient on the forehead, feel an irregular pulse. Yes, it has something to do with the clinical gaze, look at the patient… is the patient pale … you use all your senses’ (Langkjaer et al. 2021, 1793) Subthemes—Clinical gaze, senses, experience versus inexperience, intuition	Intuition
Exemplar 3: Bingham et al. (2020)	Communication, teamwork, knowledge, workload, situation awareness, intuition, working memory, clinical judgement, organisational culture	‘I think the main thing's, probably, previous experience. Because it happens quite frequently on this ward, most of the time you know what sort of patients are going to deteriorate in certain ways’ (Bingham et al. 2020, 2175) Subthemes—Inexperience versus experience, teamwork, clinical judgement	Effective teams
Exemplar 4: Orique et al. (2019)	Situational awareness clinical judgement, knowing the patient	‘Tendency to classify cues as signals was significantly (*p* < 0.05) different across medical‐surgical subspecialties. Specifically, nurses working in the acute postsurgical unit had a significantly (*p* < 0.05) lower tendency to classify patient cues as signals of clinical deterioration compared with nurses working in renal, oncology and observation units. In the immediate postoperative period, variability in patient vitals may lead nurses to use criteria that are more restrictive in identifying clinical deterioration’ Sub theme—less restrictive observation requirements enabled nurses an opportunity to know the patient, have situational awareness and use clinical judgement	Situational awareness

### 2.3 Results

Twenty‐four papers were included in the review. Eighteen adopted qualitative methodology, four were quantitative studies and two used mixed methods. The studies were carried out across nine countries: nine in the USA, six in Australia, three in Denmark and one each in Finland, Singapore, the UK, Ireland, Indonesia and Italy. Nurses were participants in all studies, however, some studies included other participants such as medical officers or medical specialists (Chua et al. 2020; Flenady et al. 2020; Garza et al. 2021; Gawronski et al. 2018; Mølgaard et al. 2022; Saunders et al. 2021; Walker et al. 2021), midwives (Mikhail and King 2025), health care workers (Foley and Dowling 2019; Mikhail and King 2025; Smith et al. 2021), parents of paediatric patients (Gawronski et al. 2018) and patients (McNeill et al. 2019; Walker et al. 2021). Table 6 details the characteristics of included studies.

**TABLE 6:** Characteristics of included studies.

Authors	Year	Country	Methodology	Design	Participants	Data collection methods
Anton et al.	2021	USA	Qualitative	Qualitative	Nurses	Focused interviews
Astroth et al.	2013	USA	Qualitative	Qualitative	Nurses	Semi‐structured interviews
Azimirad et al.	2021	Finland	Quantitative	Descriptive correlational	Nurses	Questionnaires
Bingham et al.	2020	Australia	Qualitative	Exploratory descriptive	Nurses	Structured interviews
Burns et al.	2018	USA	Qualitative	Phenomenology	Nurses	Semi‐structured interviews
Chua et al.	2020	Singapore	Qualitative	Grounded theory	Nurses, Medical officers	Semi‐structured interviews
Ekwantoro et al.	2020	Indonesia	Quantitative	Analytic correlation/cross‐sectional	Nurses	Questionnaires
Flenady et al.	2020	Australia	Qualitative	Interpretive	Nurses, Medical officers	Semi‐structured interviews
Foley & Dowling	2019	Ireland	Qualitative	Holistic single case study	Nurses, Health Care Assistants	Semi‐structured interviews
Garza et al.	2021	USA	Qualitative	Qualitative	Nurses, Medical specialists	Semi‐structured interviews
Gawronski et al.	2018	Italy	Qualitative	Qualitative	Nurses, Medical officers, parents of paediatric patients	Semi‐structured interviews
Hart et al.	2014	USA	Quantitative	Analytic correlation/cross‐sectional	Nurses	Questionnaires
Langkjaer et al.	2021	Denmark	Mixed methods	Exploratory descriptive	Nurses	Focus groups
Massey et al.	2013	Australia	Qualitative	Interpretive	Nurses	Semi‐structured interviews
McNeill et al.	2019	USA	Mixed methods	Mixed methods	Nurses, Patients	Focus groups, Questionnaires
Mikhail & King	2025	Australia	Qualitative	Interpretive descriptive	Nurses, midwives, enrolled nurses, nursing assistants	Semi‐structured interviews
Molgaard et al.	2022	Denmark	Qualitative	Ethnography	Nurses, Medical officers	Focused interviews, Participation observation
Orique et al.	2019	USA	Quantitative	Analytic correlation/cross‐sectional	Nurses	Questionnaires
Petersen et al.	2017	Denmark	Qualitative	Prospective observational cohort study	Nurses	Focus groups
Ruiz et al.	2024	USA	Qualitative	Descriptive	Nurses	Semi structured interviews
Saunders et al.	2021	Australia	Qualitative	Observational	Nurses, Medical officers	Semi‐structured interviews
Smith et al.	2021	United Kingdom	Qualitative	Ethnography	Nurses, Health Care Workers	Semi‐structured interviews
Thrasher et al.	2017	USA	Qualitative	Qualitative	Nurses	Structured interviews
Walker et al.	2021	Australia	Qualitative	Grounded theory	Nurses, Medical officers, Patients, Allied Health	Semi‐structured interviews

#### 2.3.1 Findings of the Review

Six themes were derived from the synthesis. The distribution of studies synthesised to each theme is presented in Table 7.

**TABLE 7:** Theme by study matrix.

Study	Situational awareness	Teamwork and communication	Leadership and culture	Intuition	Experience versus inexperience	Fear of retribution
Anton et al. (2021)	✓	✓		✓	✓	✓
Astroth et al. (2013)			✓		✓	✓
Bingham et al. (2020)	✓	✓	✓	✓	✓	
Burns et al. (2018)	✓			✓		
Chua et al. (2020)		✓	✓	✓		✓
Ekwantoro and Rusca (2020)		✓			✓	
Flenady et al. (2020)		✓	✓		✓	
Foley and Dowling (2019)				✓	✓	
Garza et al. (2021)	✓	✓	✓		✓	
Gawronski et al. (2018)	✓	✓			✓	
Hart et al. (2014)			✓		✓	
Langkjaer et al. (2021)	✓			✓	✓	✓
Massey et al. (2013)		✓	✓		✓	✓
McNeill et al. (2019)		✓				
Mikhail and King (2025)	✓	✓	✓		✓	
Mølgaard et al. (2022)	✓					
Orique et al. (2019)	✓				✓	
Petersen et al. (2017)		✓			✓	
Ruiz et al. (2024)	✓	✓	✓	✓	✓	✓
Saunders et al. (2021)	✓	✓	✓		✓	
Smith et al. (2021)				✓	✓	✓
Thrasher et al. (2017)		✓	✓		✓	✓
Walker et al. (2021)	✓	✓	✓	✓	✓	✓
Total	12	15	13	9	20	9

*Note:* Theme frequency reflects the number of studies contributing data to each theme and should not be interpreted as an indicator of the relative importance or clinical significance of any theme. All six themes are considered equally meaningful contributions to the synthesis.

##### 2.3.1.1 Theme One: The Importance of Situation Awareness

Seven studies discussed the importance of situational awareness in recognising clinical deterioration in patients (Anton et al. 2021; Bingham et al. 2020; Burns et al. 2018; Garza et al. 2021; Langkjaer et al. 2021; Mølgaard et al. 2022; Saunders et al. 2021). From these studies, situational awareness was described as more than responding to visual cues. Situational awareness was the ability to be able to predict a likely outcome of the deteriorating patient due to the nurse's presence with the patient, utilising their working memory and knowledge of the situation to be able to correctly inform decision making.

Situational awareness, in particular working memory, was sometimes termed ‘clinical gaze’ and allowed nurses to develop and use all of their non‐technical skills and to trust their gut feeling based on a smell or a touch, alerting them to potential clinical deterioration. This working memory is developed over years, informed by past individual experiences and training, and prompted by the senses to create an environment that draws on insight whilst simultaneously prioritising, delegating and informing clinical decision making (Langkjaer et al. 2021; Saunders et al. 2021). Langkjaer et al. (2021) describe not simply relying on the technical skill of vital signs or an early warning scoring system. ‘Just to touch and feel the patient on the forehead, feel an irregular pulse… yes, it has something to do with the clinical gaze, look at the patient… you use all your senses when you assess the patient’ (Langkjaer et al. 2021, 1793). Such practice leads to increased awareness and ability to predict patient outcomes.

Experienced nurses use their working memory to give precedence to critical information, categorising relevant information from their memory to assist with clinical decision making (Anton et al. 2021; Burns et al. 2018). Working memory for nurses is an acquired non‐technical skill where nurses not only work with memory but with current information without losing track of what they are already doing, using visual cues to expect the unexpected, being able to use foundational knowledge and experience when predicting a patient's deterioration or outcome.

In addition, the nurses' ability to be present is an important situational awareness skill, as time spent with patients allows for critical thinking and decision making to further enhance the clinical gaze. This is a result of nurses having opportunities to observe the whole patient, looking for any changes including behavioural, compiling and categorising information and close monitoring of the whole patient status that may lead to early detection of a deterioration allowing for quick response (Anton et al. 2021; Bingham et al. 2020). ‘Being with a patient face to face, I can assess them no matter what I am doing. Just being person to person, it's an important thing….’ (Anton et al. 2021, 15).

##### 2.3.1.2 Theme Two: Effective Teamwork and Communication

Nine studies reported the finding that teamwork and collaboration are critical non‐technical skills in the recognition and response to clinical deterioration. Effective communication within the team was required to utilise clinical judgement in whether to escalate due to suspected clinical deterioration or not (Anton et al. 2021; Bingham et al. 2020; Garza et al. 2021; Orique et al. 2019; Petersen et al. 2017; Ruiz et al. 2024; Walker et al. 2021). Garza et al. (2021) proposed that ‘teamwork’ was the reason nurses had positive perceptions of care delivery during episodes of clinical deterioration.

However, there are some distinct barriers to effective teamwork and communication. Barriers include the business of the unit, the hierarchy of doctor‐nurse relationships and inexperience of nurses (Bingham et al. 2020; Chua et al. 2020; Gawronski et al. 2018; Ruiz et al. 2024; Saunders et al. 2021). The progression from recognition to responding to (potential) clinical deterioration and management of deterioration involves the nurse collating their findings and communicating the information to the team including the doctor. If the unit is busy, or the nurse is inexperienced, they may miss the deterioration or not clearly communicate, which will result in a delayed response. Ineffective communication styles included team members who were aggressive or abrupt, which left some nurses feeling unsupported in being able to effectively communicate the patient situation, therefore impacting their ability to respond to the deterioration (Saunders et al. 2021; Astroth et al. 2013). ‘So (teamwork) kind of ties into everything else. So having a leader who is communicating well, having a team who's communicating well…’ (Saunders et al. 2021, 92). ‘… so they can paint a little bit of a picture’ (Ruiz et al. 2024, 5).

Another barrier to effective communication is workload, including staff shortages, time constraints and cognitive overload due to the fast‐paced nature of acute clinical units. ‘Because we do not speak or have time to speak’ (Gawronski et al. 2018, 5). ‘…. sometimes I feel like you're so frazzled after a shift that you're like “I have no idea”’ (Walker et al. 2021). Poor workload management played a part in ineffective teamwork, therefore lessening collaboration, especially among the inexperienced/novice nurses. Experienced nurses were found to be better able to prioritise tasks and recognise and respond to a clinical deterioration by using the clinical gaze and pulling information from their working memory. However, due to the high workload within units, they were unable to assist more junior nurses (Anton et al. 2021; Bingham et al. 2020). ‘I think the main thing's, probably, previous experience. Because it happens quite frequently on this ward, most of the time you know what sort of patients are going to deteriorate in certain ways’ (Bingham et al. 2020, 2175).

The hierarchy of doctor‐nurse relationship can be a contributing barrier to collaborative teamwork, especially if the doctor responding is a junior doctor or the nurse is a novice/inexperienced nurse, resulting in ineffective communication especially in the absence of quantifiable evidence of the deterioration (Chua et al. 2020, 58). Petersen et al. (2017) expressed that nurses were more inclined to communicate with doctors if they had a good relationship and were experienced but were reluctant to call junior doctors as the nurses felt the junior doctors were inexperienced, and their contributions were not helpful.

##### 2.3.1.3 Theme Three: Leadership and Culture

Non‐technical skills include the use of cognitive, social and personal experience skills which, when combined, establish leadership (Walker et al. 2021). This is especially important when trying to establish teamwork that encourages a culture of safe and efficient patient care. The development of non‐technical skills enhances nurses' ability to apply effective communication, especially in leadership roles (Walker et al. 2021). A lack of leadership has been found to cause frustration or a negative culture of distrusting those in leadership roles and concern regarding the repercussions or stigma associated with escalating a deterioration call. ‘… the junior nurses… escalated the concern and the senior nurse has shot them down and said “No. You don't have to worry about that”’ (Walker et al. 2021, 810). ‘I think some nurses, there's a stigma about calling a rapid. Some people don't want to because they're worried about what the ICU nurse is gonna say to them’ (Ruiz et al. 2024, 4).

Alternatively, a positive culture ensues when the leadership team encourages positive work behaviours and effective communication, ensuring a culture of safe, quality care (Astroth et al. 2013; Hart et al. 2014; Orique et al. 2019; Walker et al. 2021). Hart et al. (2014) describe leadership as nurses having self‐confidence in being able to recognise and respond to a deterioration, calling on strong clinical reasoning and decision‐making skills during the patient deterioration to effect positive patient outcomes. This was further demonstrated in Astroth and colleague's study: ‘As a charge nurse I try to encourage anyone to call, including aides…’ (Astroth et al. 2013, 2879). Mikhail & King described collegial support and mentorship which is a trait in leadership. ‘Take them aside privately. Have some one‐to‐one chats, try to do a bit of mentoring, find out areas of weaknesses that we could work on together. Provide a feedback time, check back time. Find out if we could do something together*’* (Mikhail and King 2025, 7).

This form of leadership speaks to empowering a culture of ‘all in the team’ having a role in the detection and response to deterioration and not just those in assigned leadership roles or expertise. This leadership also extended to the rapid response team ‘“Why did you call? This wasn't necessary” …. Once a nurse gets attitude (from the rapid response team members), they don't want to call again’ (Astroth et al. 2013, 2879).

##### 2.3.1.4 Theme Four: Intuition

Eleven papers described senior nurses use of intuition, or ‘knowing the patient’ when recognising an episode of clinical deterioration. Experienced nurses' intuition inspired them to trust their own judgement as a facet of preparedness (Astroth et al. 2013; Bingham et al. 2020; Chua et al. 2020; Ekwantoro and Rusca 2020; Foley and Dowling 2019; Gawronski et al. 2018; Langkjaer et al. 2021; Mikhail and King 2025; Ruiz et al. 2024; Smith et al. 2021; Walker et al. 2021). Bingham et al. (2020) concluded that knowing the patient allows the nurse to sense there is something wrong. This demonstrates the importance of nurses using their perception as a non‐technical skill especially when the patient's vital signs are within normal range and not meeting escalation criteria. The nurses' experience enhances the intuitive decision‐making process, which is both cognitive and experiential, in being able to see what is not overt. This demonstrates the non‐technical expertise of senior nurses, which unfortunately novice nurses may struggle to conceptualise due to their inexperience as they primarily rely on their ability to recognise the physiological signs of deterioration. This is captured in the following exemplars:

It's not just the numbers. You can look at your patient and you …. just know. (Walker et al. 2021, 810)

Nurses don't go with everything on the protocol, they (sic) do use their knowledge and experience. (Foley and Dowling 2019, 1186)

The clinical gaze needs to be trained. You do not have it when you graduate from nursing school… and it takes a certain number of years before you get it. (Langkjaer et al. 2021, 1793)

You're given an edge to have like that ‘clinical eye’. So, you can even see it before it's happening. So, I don't think you get that from books you know, you get that from years of experience. (Mikhail and King 2025, 6)

##### 2.3.1.5 Theme Five: Experience Versus Inexperience

Experienced nurses have the ability to draw on their non‐technical skills which enhances their decision‐making skills, while inexperienced nurses lacking these non‐technical skills impede their clinical judgement and decision‐making (Gawronski et al. 2018). A senior nurse's ability to recall past experiences supports clinical judgement thus facilitating escalation of care; however, peer‐to‐peer mentoring would give inexperienced nurses the ability to gain knowledge and make clinical judgements with support. ‘I think that the main thing in question, self‐doubt … is the patient really sick enough to call?’ (Astroth et al. 2013, 2879).

Experienced nurses do not rely solely on monitoring equipment or technical assessment to detect deterioration, rather they admit to knowing the importance of carrying out a thorough assessment. They use a wide variety of non‐technical skills, however, the less experienced or novice nurses are generally unable to realise the significance of subtle changes in a patient's behaviour or appearance due to lack of experience or ‘knowing their patients’ (Anton et al. 2021; Ruiz et al. 2024). ‘I think that's (observational skills) what makes nurses good nurses … I don't even necessarily have to have the numbers’ (Anton et al. 2021, 6). ‘I think it's just experience. Just working and just gaining confidence through experiences’ (Mikhail and King 2025, 6). Experienced nurses, compared to inexperienced nurses, can also draw on past experiences to gain knowledge around what to expect or what may happen, building confidence in their ability to detect a deterioration, ‘You start…at your level with your peer or your charge nurse and then you escalate’ (Ruiz et al. 2024, 4). A lack of early detection of subtle changes may result in a lack of rigorous assessment and result in progression of patient deterioration (Astroth et al. 2013; Ekwantoro and Rusca 2020; Gawronski et al. 2018; Massey et al. 2013; Mikhail and King 2025; Orique et al. 2019).

I think it's probably a lack of understanding of the MET (medical emergency team) and how it should be used. People don't see it as an early intervention thing. (Massey et al. 2013, 135)

##### 2.3.1.6 Theme Six: Fear of Retribution

Part of the process of escalating a deterioration is the nurse at the bedside being able to recognise the deterioration and knowing how to communicate this without fear of retribution for not having all of the information or fully understanding the deterioration.

‘I just need someone of seniority to back me up when the doctor scolds me for activating a MET (medical emergency team)’ (Chua et al. 2020, 57). Likewise, Walker et al. (2021) and Ruiz et al. (2024) found:

…. fear of looking stupid, and the fear of not being able to deal with it themselves… they are seen to have to be able to handle whatever comes their way. (Walker et al. 2021, 810)

Oh, your patient vasovagaled? Are they fine now? Okay, goodbye. We're not gonna take them anyway…So why'd you call? (Ruiz et al. 2024. 4)

If nurses don't feel validated for escalating a concern, they begin to question their capability, ‘Maybe questioning my decisions: Am I overreacting here? Is this real or am I just panicking?…Just feeling I'd better not do that kind of thing or I might get told off’ (Massey et al. 2013, 135).

The use of non‐technical skills such as teamwork, collaboration, leadership, intuition and communication is necessary, yet nurses continue to experience fear of retribution for escalating clinical deterioration. Being reprimanded and feeling stupid is a common denominator for delayed escalation of patient deterioration. Langkjaer et al. (2021) argue that nurses use non‐technical skills along with technical assessment skills when recognising and responding to patient deterioration. However, this may create a culture where nurses are fearful of making mistakes or where their non‐technical skills and assessments are undermined. ‘I think that some newer nurses are reluctant to call it because…do you want everyone to think you don't know what you're doing?’ (Astroth et al. 2013, 2879).

The fear of being reprimanded by other team members elicits a negative and emotional response in nurses, reducing their coping mechanisms. This negative culture creates emotional responses in nurses such as shame or rejection, creating moral distress. This is particularly evident in novice nurses, where there are professional role expectations, time constraints and lack of knowledge and decision‐making ability, perpetuating fear and anxiety thereby resulting in a delay to detect or respond to a deteriorating patient (Chua et al. 2020; Langkjaer et al. 2021; Walker et al. 2021; Massey et al. 2013). ‘Nurses feel like they are going to be told off for wasting the medical emergency team's time. Even though worried or concerned is in the little cards we all carry around’ (Massey et al. 2013, 135). However, Astroth et al. (2013), recognised that a unit culture of teamwork and willingness to support each other and listen to each other encourages trust around being able to make clinical decisions.

## 3 Discussion

This systematic review identified six overarching themes that characterise nurses' non‐technical skills in recognising and responding to clinical deterioration in hospital settings. The findings reveal that situational awareness formed the cornerstone of effective deterioration recognition, enabling nurses to perceive, interpret and act on subtle changes in patient condition. Effective teamwork and communication emerged as essential to timely escalation, yet were frequently undermined by hierarchical structures and high workload. Leadership and organisational culture shaped the extent to which nurses felt empowered to act, while intuition grounded in clinical decision making and experience played a significant role in prompting early intervention. In contrast, limited level of experience constrained nurses' capacity to recognise deterioration and communicate concerns effectively, with fear of retribution emerging as a critical barrier to escalation, particularly among junior nurses. Together, these themes illuminate the complex interplay of individual, team and organisational factors that influence nurses' social and cognitive skills (non‐technical skill) and therefore performance in responding to clinical deterioration. Effective teamwork and communication rely on clinical judgement to detect and arrest patient deterioration. However, some barriers to effective team performance and productive communication exist such as high workload within units, the hierarchy of doctor‐nurse relationships and the inexperience of nurses. McGraw (2019) concurs that lack of knowledge and experience in recognising and responding to clinical deterioration decreases the opportunity for new or inexperienced nurses to monitor their patients for any changes in their condition. However, it is the relationship aspect of being a new or inexperienced nurse in terms of teamwork and communication which may cause clinical deterioration to be unrecognised. Additionally, the perceived hierarchy between doctor and nurse in the multidisciplinary team has been recognised as a barrier to teamwork, decreasing nurses' capability to communicate about clinical deterioration in a non‐collaborative environment (Chua et al. 2020; Walker et al. 2021). Nurse's workload is acknowledged in the literature as a significant barrier to effective communication practices, particularly among inexperienced nurses, who may be unable to prioritise their duties in responding to clinical deterioration. The nursing workload can prevent prompt detection of deterioration, which can be considered as the barrier to the escalation of healthcare (Brady and Goldenhar 2014).

Intuition or ‘knowing the patient’ was perceived as an important non‐technical skill for nurses in recognising and responding to any changes in a patient's condition. Intuition coupled with clinical knowledge could allow nurses to depict the patients' recovery journey in their minds and predict changes, which may assist them to recognise and respond to clinical deterioration. This is echoed by a Brier et al. (2015) study concluding that nurses' intuition was considered as an intuitive knowing which allows them to use tacit knowledge of their past experiences to recognise and respond at the early stages of deterioration, thus minimising poor outcomes. A similar study also highlighted that such intuition can be used as a helpful tool for nurses to provide additional support for their patients by anticipating clinical deterioration, intervening early and preventing further severe symptoms and adverse outcomes (Dalton et al. 2018).

Having previous experiences of patient deterioration allows nurses to measure, interpret and respond to clinical observations accurately and subsequently manage deterioration events better, compared to those who have not. Past and vast experiences are considered significant factors in helping nurses to present a practical assessment and rescue patients when signs and symptoms go unrecognised (Smith et al. 2021). This past and vast experience is impacted by the theme of fear of retribution. Particularly fear of retribution captures the unique dynamic where nurses hesitate to call for help due to concerns about team perceptions. Fear of retribution by the members of the rapid response team or colleagues was also considered a potential factor that may prevent nurses from escalating clinical deterioration. Such fear is more prevalent in inexperienced nurses who may lack knowledge, confidence and communication skills, thus creating a climate where escalating clinical deterioration is less likely to happen. Furthermore, fear of being criticised as having insufficient clinical experience and inadequate knowledge often results in seeking assistance or affirmation of information or decision making, leading to delays in recognising and responding to clinical deterioration (O'Neill et al. 2021).

Effective nursing leadership fosters self‐confidence in using clinical skills and leadership abilities to respond and intervene with the appropriate actions. Strong leadership skills play an integral role in helping nurses act promptly and quickly and execute in a coordinated, efficient manner to recognise and respond to clinical deterioration (Massey et al. 2013). Also, adopting effective leadership allows nurses to embed a positive culture that empowers and motivates them to support less experienced nurses to recognise when to respond to clinical deterioration appropriately, without fear. Leadership with a positive culture can motivate nurses to focus on identifying and managing clinical deterioration and envisioning better outcomes by mitigating unnecessary delays and facilitating provision of high‐level care, simultaneously strengthening their confidence (Azimirad et al. 2022).

## 4 Limitations

While quality appraisal did not indicate systemic weaknesses warranting exclusion, studies were not weighted by quality. This is acknowledged as a limitation, with sensitivity analysis suggested for future reviews. Despite comprehensive search strategies, the review may be subject to publication bias, as studies with non‐significant findings are less likely to be published.

In addition, the authors acknowledge the complexity of separating the non‐technical attributes in the recognition of, and response to, clinical deterioration from what has traditionally been understood in the literature, in training and in education as a technical skill or knowledge. Understanding recognition and response to clinical deterioration, is both a technical and non‐technical skill.

## 5 Implications for the Profession and Patient Care

This manuscript provides an updated review of published studies since 2012 through convergent integrated synthesis, and demonstrates that recognition and response to clinical deterioration, even in the presence of well‐established early warning systems, are dependent on nurses' non‐technical skills (situational awareness, communication, teamwork, leadership, intuition) and supportive organisational cultures. The results present an extension of Peddle et al.'s (2018) work by synthesising how non‐technical skills interact specifically in clinical deterioration contexts, including facilitators, constraints and acute care challenges. Future research should seek to develop a new conceptual framework positing the interdependence between technical and non‐technical skills in the recognition of, and response to, clinical deterioration. This review provides a robust empirical foundation for such developments.

Nursing curricula should incorporate simulation‐based training in recognising subtle deterioration cues, structured escalation communication and psychological safety in teams. In practice, clinical organisations must facilitate psychologically safe cultures by implementing mentoring, bedside coaching and non‐punitive debriefing, with particular emphasis on junior or new nurses, to strengthen non‐technical skills of staff in recognising and responding to clinical deterioration. Policy recommendations for clinical organisations include integrating non‐technical skills into deterioration response frameworks, workforce development strategies and patient safety standards alongside rapid response systems to align organisational processes with evidence on effective escalation.

## 6 Conclusion

The results of this study have enabled the team to identified and highlighted the importance of non‐technical skills in the recognition and response to the deteriorating patient. Though non‐technical skills such as situational awareness, communication, teamwork and leadership are well reported in the literature, they have not been corroborated together as a significant barrier to the effective recognition of, and response to, clinical deterioration. Likewise, high workloads within units, the hierarchy of doctor‐nurse relationships and experience of nurses are barriers to effective communication and teamwork, essential in the recognition of, and response to, deterioration. A positive workplace culture and supportive leadership foster effective communication and promote early detection and response within the context of hospitals; therefore, valuing nurses' non‐technical skills is required by the entire healthcare team.

The implementation of early warning scores (EWS) or rapid response systems (RRS) is a well‐developed mechanism to improve outcomes for deteriorating patients. The findings of this systematic review reveal the value and importance placed on non‐technical skills and to effectively recognise and respond to clinical deterioration greater emphasis is needed to be given to non‐technical skills in the context of rapid response systems.

## Author Contributions

Darlene Archer: conceptualisation, data curation, formal analysis, writing – original draft, writing – review and editing. Amineh Rashidi: conceptualisation, data curation, supervision, validation, writing – original draft, writing – review and editing. Rajbeer Kaur: conceptualisation, data curation, validation, writing – original draft, writing – review and editing. Allison Roderick: data curation, formal analysis, writing – original draft, writing – review and editing. Olivia Gallagher: data curation, methodology, project administration, supervision, validation, visualisation, writing – original draft, writing – review and editing.

## Funding

The authors have nothing to report.

## Conflicts of Interest

The authors declare no conflicts of interest.

## Supporting information


**File S1:** PRISMA 2020 checklist.

**File S2:** Search strings.

**File S3:** Quality appraisal.

## Data Availability

Data sharing not applicable to this article as no datasets were generated or analysed during the current study.
