# Supplementary material for: Nurses' Perceptions of Their Non‐Technical Skills in Recognising and Responding to Clinical Deterioration in Hospital Settings: A Systematic Review
Source: J Clin Nurs. 2026 Aug 13;35(12):5458–72. doi: 10.1111/jocn.70497 (PMC13744222; doi:10.1111/jocn.70497)
Supplement: Supplementary file 1 [file JOCN-35-5458-s001.docx]

**Supplementary File 3**

**Quality Appraisal**

| **Citation** | **Appraisal Tool** | **Q1** | **Q2** | **Q3** | **Q4** | **Q5** | **Q6** | **Q7** | **Q8** | **Q9** | **Q10** |
| --- | --- | --- | --- | --- | --- | --- | --- | --- | --- | --- | --- |
| Anton et al. 2021 | Qualitative | U | Y | Y | Y | Y | N | N | Y | Y | Y |
| Astroth, 2013 | Qualitative | Y | Y | Y | Y | Y | N | Y | Y | Y | Y |
| Azimirad et al. 2021 | Analytical cross sectional | Y | Y | Y | Y | NA | NA | Y | Y | - | - |
| Bingham et al. 2020 | Qualitative | Y | Y | y | Y | Y | NA | Y | Y | Y | Y |
| Burns et al. 2018 | Qualitative | Y | Y | Y | Y | Y | NA | U | Y | Y | Y |
| Chua et al. 2020 | Qualitative | Y | Y | Y | Y | Y | NA | Y | Y | Y | Y |
| Ekwantoro et al. 2020 | Analytical cross sectional | Y | Y | N | U | Y | Y | Y | Y | - | - |
| Flenady et al. 2020 | Qualitative | Y | Y | Y | Y | Y | NA | Y | Y | Y | Y |
| Foley & Dowling, 2019 | Qualitative | Y | Y | Y | Y | Y | NA | NA | Y | Y | Y |
| Garza et al. 2021 | Qualitative | Y | Y | Y | Y | Y | NA | Y | Y | Y | Y |
| Gawronski et al. 2018 | Qualitative | Y | Y | Y | Y | Y | N | Y | Y | Y | Y |
| Hart & Spiva, 2014 | Analytical cross sectional | Y | Y | Y | Y | Y | Y | Y | Y | Y | Y |
| Langkjaer et al. 2021 | Qualitative | Y | Y | Y | Y | Y | N | Y | Y | Y | Y |
| Massey, 2014 | Qualitative | Y | Y | Y | Y | Y | Y | Y | Y | Y | Y |
| McNeill et al. 2019 | MMAT | Y | Y | Y | Y | Y | Y | Y | C | Y | Y |
| Mikhail & King, 2025 | Qualitative | Y | Y | Y | Y | Y | N | Y | Y | Y | Y |
| Molgaard et al. 2022 | Qualitative | Y | Y | Y | Y | Y | Y | Y | Y | Y | Y |
| Orique et al. 2019 | Analytical cross sectional | Y | Y | Y | y | Y | Y | Y | Y | Y | Y |
| Petersen et al. 2017 | Qualitative | Y | Y | Y | Y | Y | Y | Y | Y | N | Y |
| Ruiz et al., 2024 | Qualitative | U | Y | Y | Y | Y | N | Y | Y | Y | Y |
| Saunders et al., 2021 | Analytical cross sectional | Y | Y | Y | U | Y | Y | Y | Y | - | - |
| Smith et al., 2021 | Qualitative | Y | Y | Y | Y | Y | Y | Y | Y | Y | Y |
| Thrahser et al. 2017 | Qualitative | Y | Y | Y | U | Y | Y | Y | Y | Y | Y |
| Walker et al. 2021 | Qualitative | Y | Y | Y | Y | Y | Y | Y | Y | Y | Y |

Y=yes, N=no, U=unclear, C= can’t tell, NA=not appliable
