# Supplementary material for: Nurses' Perceptions of Their Non‐Technical Skills in Recognising and Responding to Clinical Deterioration in Hospital Settings: A Systematic Review
Source: J Clin Nurs. 2026 Aug 13;35(12):5458–72. doi: 10.1111/jocn.70497 (PMC13744222; doi:10.1111/jocn.70497)
Supplement: Supplementary file 3 [file JOCN-35-5458-s003.docx]

**Supplementary File 2**

**Search strings**

**Databases:** MEDLINE, Embase, PsycINFO, CINAHL and Scopus.

MEDLINE

All keywords searched as .mp (headings or sub-headings not used to explode)
Nurs* OR Enrolled nurs* OR Registered nurs*

AND

Non-clinical skills OR Non-technical skills OR Situational awareness OR Decision making OR Teamwork OR Leadership OR Communication OR Managing stress OR Coping with fatigue

AND

Acute care OR In-patient OR Hospital OR Health service OR Health care facility OR Tertiary

AND

Perception OR Belief OR Experience OR Outcome

Limit to English

Limit to Full Text

Limit to 2022 – Current

EMBASE

All keywords searched as .mp (headings or sub-headings not used to explode)
Nurs* OR Enrolled nurs* OR Registered nurs*

AND

Non-clinical skills OR Non-technical skills OR Situational awareness OR Decision making OR Teamwork OR Leadership OR Communication OR Managing stress OR Coping with fatigue

AND

Acute care OR In-patient OR Hospital OR Health service OR Health care facility OR Tertiary

AND

Perception OR Belief OR Experience OR Outcome

Limit to English

Limit to Full Text

Limit to 2022 – Current

PSYCHINFO

All keywords searched as .mp (headings or sub-headings not used to explode)
Nurs* OR Enrolled nurs* OR Registered nurs*

AND

Non-clinical skills OR Non-technical skills OR Situational awareness OR Decision making OR Teamwork OR Leadership OR Communication OR Managing stress OR Coping with fatigue

AND

Acute care OR In-patient OR Hospital OR Health service OR Health care facility OR Tertiary

AND

Perception OR Belief OR Experience OR Outcome

Limit to English

Limit to Full Text

Limit to 2022 – Current

CINAHL

All keywords searched as .mp (headings or sub-headings not used to explode)
Nurs* OR Enrolled nurs* OR Registered nurs*

AND

Non-clinical skills OR Non-technical skills OR Situational awareness OR Decision making OR Teamwork OR Leadership OR Communication OR Managing stress OR Coping with fatigue

AND

Acute care OR In-patient OR Hospital OR Health service OR Health care facility OR Tertiary

AND

Perception OR Belief OR Experience OR Outcome

Limit to English

Limit to Full Text

Limit to 2022 – Current

SCOPUS

All keywords searched as .mp (headings or sub-headings not used to explode)
Nurs* OR Enrolled nurs* OR Registered nurs*

AND

Non-clinical skills OR Non-technical skills OR Situational awareness OR Decision making OR Teamwork OR Leadership OR Communication OR Managing stress OR Coping with fatigue

AND

Acute care OR In-patient OR Hospital OR Health service OR Health care facility OR Tertiary

AND

Perception OR Belief OR Experience OR Outcome

Limit to English

Limit to 2022 – Current
